# Antidyslipidemic potential of a novel farnesoid X receptor antagonist in a hamster model of dyslipidemia: Comparative studies of other nonstatin agents

**DOI:** 10.1002/prp2.390

**Published:** 2018-03-08

**Authors:** Emiko Shinozawa, Yuichiro Amano, Hiroko Yamakawa, Megumi Haba, Mitsuyuki Shimada, Ryuichi Tozawa

**Affiliations:** ^1^ Research Takeda Pharmaceutical Company Limited Fujisawa Kanagawa Japan

**Keywords:** cholestyramine, compound‐T1, dyslipidemic hamster model, ezetimibe, FXR antagonist, torcetrapib

## Abstract

We attempted to clarify the therapeutic capability of antagonists of the farnesoid X receptor (FXR), a nuclear receptor that regulates lipid and bile acid metabolism. Herein, we report the antidyslipidemic effects of a novel synthesized FXR antagonist, compound‐T1, utilizing a dyslipidemic hamster model. Compound‐T1 selectively inhibited chenodeoxycholic acid‐induced FXR activation (IC_50_, 2.1 nmol·L^−1^). A hamster model of diet‐induced hyperlipidemia was prepared to investigate the antidyslipidemic effects of compound‐T1 through comparative studies of the nonstatin lipid‐modulating agents ezetimibe, cholestyramine, and torcetrapib. In the hamster model, compound‐T1 (6 mg·kg^−1^·day^−1^, p.o.) increased the level of plasma high‐density lipoprotein (HDL)‐cholesterol (+22.2%) and decreased the levels of plasma non‐HDL‐cholesterol (−43.6%) and triglycerides (−31.1%). Compound‐T1 also increased hepatic cholesterol 7α‐hydroxylase expression and fecal bile acid excretion, and decreased hepatic cholesterol content. Moreover, the hamster model could reflect clinical results of other nonstatin agents. Torcetrapib especially increased large HDL particles compared with compound‐T1. Additionally, in the human hepatoma Huh‐7 cells, compound‐T1 enhanced apolipoprotein A‐I secretion at a concentration close to its IC_50_ value for FXR. Our results indicated the usefulness of the hamster model in evaluating FXR antagonists and nonstatin agents. Notably, compound‐T1 exhibited beneficial effects on both blood non‐HDL‐cholesterol and HDL‐cholesterol, which are thought to involve enhancement of cholesterol catabolism and apolipoprotein A‐I production. These findings aid the understanding of the antidyslipidemic potential of FXR antagonists with a unique lipid and bile acid modulation.

**Abbreviations**
C4: 7α‐hydroxy‐4‐cholesten‐3‐one
CDCA: chenodeoxycholic acid
CETP: cholesteryl ester‐transfer protein
CYP7A1: cholesterol 7α‐hydroxylase
FXR: farnesoid X receptor
HDL: high‐density lipoprotein
HRP: horseradish peroxidase
LDL: low‐density lipoprotein
LXRs: liver X receptors
PCR: polymerase chain reaction
RXRα: retinoid X receptor‐α
SHP‐1: short heterodimer partner 1
TG: triglyceride
VLDL: very low‐density lipoprotein

## 1 INTRODUCTION

High blood cholesterol is a risk factor for cardiovascular events.^1^, ^2^ 3‐Hydroxymethyl 3‐glutaryl‐CoA reductase inhibitors, also known as statins, markedly reduce both blood cholesterol and cardiovascular risk in hyperlipidemia patients.^3^, ^4^, ^5^ However, for patients who cannot achieve their cholesterol‐lowering targets with statins, owing to insufficient efficacy or adverse effects, nonstatin agents are also effective as an alternate or add‐on therapy to statins.^6^

One such nonstatin agent is cholestyramine, a bile acid sequestrant, which forms a complex with bile acids in the intestine and is then eliminated into feces. Through the augmentation of bile acid synthesis and cholesterol catabolism, cholestyramine demonstrated a significant reduction in blood cholesterol.^7^, ^8^ A recent meta‐analysis and large‐scale epidemiologic analysis suggested that the cholesterol‐lowering effects of bile acid sequestrants, including cholestyramine, may contribute to the prevention of cardiovascular diseases.^9^ Another nonstatin medication is the cholesterol absorption inhibitor ezetimibe, which targets intestinal uptake of dietary and biliary cholesterols; good efficacy and tolerability of combination therapy with statin and ezetimibe have been reported.^10^

Another approach aimed at the reduction in cardiovascular risk is the increase in blood high‐density lipoprotein (HDL)‐cholesterol levels^11^ by the inhibition of cholesteryl ester‐transfer protein (CETP), which mediates transfer of cholesteryl ester from HDLs to atherogenic lipoproteins. Clinical trials involving torcetrapib, the first CETP inhibitor, were terminated early owing to off‐target toxicity,^12^, ^13^ and despite remarkable increases in HDL‐cholesterol, torcetrapib did not reduce the progression of atherosclerosis.^14^, ^15^ The definitive clinical efficacies of CETP inhibition are under investigation in the ongoing REALIZE study of anacetrapib, a more recent CETP inhibitor.^16^

**Figure 1:**
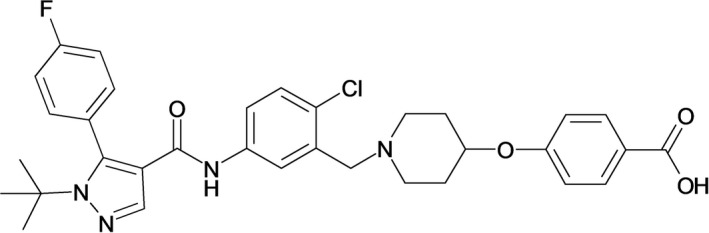
Chemical structure of compound‐T1

As a possible concept for another novel nonstatin agent, we focused on the inhibition of farnesoid X receptor (FXR), a member of the nuclear receptor superfamily. FXR is expressed at high levels in tissues involved in bile acid metabolism such as the liver, intestine, and kidney.^17^, ^18^ In the liver, FXR indirectly suppressed expression of cholesterol 7α‐hydroxylase (CYP7A1), a rate‐limiting enzyme in the cholesterol catabolic pathway,^19^ and also directly downregulated the expression of apolipoprotein A‐I, a major protein constituent of HDL.^20^ We therefore hypothesized that repression of FXR would improve dyslipidemia via both non‐HDL‐cholesterol reduction and HDL‐cholesterol elevation, and we successfully confirmed that a synthesized FXR antagonist, compound‐T3, displayed these blood changes in cynomolgus monkeys.^21^, ^22^ In this study, to clarify the therapeutic advantage of an FXR antagonist over the nonstatin agents ezetimibe, cholestyramine, and torcetrapib, we investigated pharmacological profiles of another novel synthesized FXR antagonist, 4‐({1‐[5‐({[1‐tert‐butyl‐5‐(4‐fluorophenyl)‐1H‐pyrazol‐4‐yl]carbonyl}amino)‐2‐chlorobenzyl]piperidin‐4‐yl}oxy)benzoic acid (compound‐T1, Figure 1) and conducted comparative studies in a high‐fat diet‐induced dyslipidemic hamster model.

## 2 MATERIALS AND METHODS

### 2.1 Drugs

Compound‐T1 was synthesized at Takeda Pharmaceutical Co., Ltd (Osaka, Japan). Ezetimibe was purified from commercially available tablets at Takeda Pharmaceutical Co., Ltd. Torcetrapib was synthesized at KNC Laboratories Co., Ltd (Hyogo, Japan). Cholestyramine, chenodeoxycholic acid (CDCA), and 7α‐hydroxy‐4‐cholesten‐3‐one (C4) were purchased from Sigma‐Aldrich Japan (Tokyo, Japan).

### 2.2 Nuclear receptor panel assays

The agonistic and antagonistic activities of human nuclear receptors, FXR and PPARs PPARα, PPARδ, and PPARγ were measured as previously described.^23^, ^24^ The agonistic and antagonistic activities for the human retinoid X receptor‐α (RXRα) and the human liver X receptors (LXRs) LXRα and LXRβ were also measured. Briefly, the reporter construct for the RXRα reporter gene assay, pGL3‐DR1 × 4‐tk‐luc, was created by the insertion of four copies of direct repeat 1 element (AGGTCA‐N‐AGGTCA) upstream of the herpes virus thymidine kinase promoter and the luciferase reporter gene. To construct the LXRα and LXRβ reporter gene assays, pGL3‐DR4 × 4‐tk‐luc was generated by the insertion of four copies of direct repeat 4 (AGGTCA‐NNNN‐AGGTCA) at the same position as pGL3‐DR1 × 4‐tk‐luc.

COS‐1 cells were maintained in DMEM supplemented with 10% fetal bovine serum at 37°C under 5% CO_2_. Cells were transfected in F150 flasks (Corning, NY, USA) with pMCMVneo‐hRXRα and pGL3‐DR1 × 4‐tk‐luc for RXRα assays, pMCMVneo‐hRXRα, pMCMVneo‐hLXRα, and pGL3‐DR4 × 4‐tk‐luc for LXRα assays, and pMCMVneo‐hRXRα, pMCMVneo‐hLXRβ, and pGL3‐DR4 × 4‐tk‐luc for LXRβ assays. Transfections were performed with Lipofectamine and PLUS Reagent (Thermo Fisher Scientific K.K., Kanagawa, Japan) in accordance with the manufacturer's protocol. After culture for 24 hour, cells were suspended in DMEM medium containing 0.1% fatty acid free‐BSA and plated in 96‐well white tissue culture treated plates (Corning, NY, USA) at a density of 4500 cells/well. After culture for 3 hour, the cells were treated with test compounds in the presence or absence of the respective ligand, and incubated for 1 day. The luciferase activity was measured by Envision (PerkinElmer, MA, USA) using Picagene‐LT7.5 Luminescence Reagent (Wako Pure Chemical Industries, Ltd, Osaka, Japan). The respective ligand for each receptor was considered the 100% activation control as follows: 1 μmol·L^−1^ 9‐*cis*‐retinoic acid (Wako Pure Chemical Industries, Ltd) for RXRα; 10 μmol·L^−1^ All‐*trans*‐retinoic acid (Wako Pure Chemical Industries, Ltd) for RARα; 1 μmol·L^−1^ 22R‐hydroxycholesterol for LXRα; 3 μmol·L^−1^ 22R‐hydroxycholesterol for LXRβ; 30 μmol·L^−1^ CDCA for FXR. To determine EC_50_ or IC_50_ values, the dose–response data were fitted to a four‐parameter logistic curve using GraphPad Prism (GraphPad Software, CA, USA).

### 2.3 Animals

All animal experiments were performed in accordance with the guidelines of the Takeda experimental animal care and use committee. Five‐week‐old male Golden Syrian hamsters were purchased from Japan SLC, Inc. (Shizuoka, Japan), and maintained in a room with controlled temperature and humidity conditions of 20‐26°C and 40%‐80%, respectively, under a 12‐hour light/dark cycle. The hamsters were allowed free to access to water and received a commercial standard chow diet (CE‐2; CLEA Japan Inc., Tokyo, Japan).

### 2.4 Single‐dose study

Hamsters were fed with a Western‐type diet (a chow diet containing 0.15% cholesterol and 15% nonsalt butter, Oriental Yeast Co., Ltd, Tokyo, Japan) and housed individually in cages after the age of 7 weeks. Eight‐week‐old hamsters fed with a Western‐type diet for 1 week were orally administered compound‐T1 (0.3, 1, and 3 mg·kg^−1^ in a 0.5% methylcellulose solution). Hamsters in the control group were treated with 0.5% methylcellulose vehicle solution only. The measurements of plasma C4 levels were conducted on blood samples collected from the orbital vein under nonfasting conditions at 4, 8, and 24 hour after administration (n = 6).

### 2.5 Multiple‐dose study

Hamsters were fed with a high‐fat diet (a chow diet containing 10.3% nonsalt butter, Oriental Yeast Co., Ltd) and housed individually in cages after the age of 6 weeks. After high‐fat diet feeding for 1 week, drug treatments were commenced at the age of 7 weeks. Specifically, hamsters were divided into six groups for experiment 1 (control, compound‐T1 (2 and 6 mg·kg^−1^·day^−1^), ezetimibe (0.1 and 0.3 mg·kg^−1^·day^−1^), and cholestyramine (770 mg·kg^−1^·day^−1^), n = 6), and five groups for experiment 2 (control, compound‐T1 (3 and 10 mg·kg^−1^·day^−1^), torcetrapib (30 and 100 mg·kg^−1^·day^−1^), n = 6); all groups were matched for body weight and plasma lipid levels. Food consumption was determined for each animal every day. Compound‐T1, ezetimibe, and torcetrapib were suspended in 0.5% methylcellulose solution and administered by gavage once per day for 9 days. Cholestyramine was mixed in the 10.3% butter‐containing powder diet such that the final diet contained 1% cholestyramine. Dosage was calculated from the cholestyramine content of the feed and the average food consumption during the first 7 days of drug administration. The control group was administered with a vehicle solution of 0.5% methylcellulose.

Blood samples were collected from the orbital vein using heparin as an anticoagulant on the mornings of days 1 and 8 under nonfasting conditions, which were regarded as pre‐ and post‐treatment samples, respectively, and used for measurements of the plasma lipid levels. Feces were collected from each hamster over a 48 hour period on days 6‐8 of drug administration. The hamsters were sacrificed under anesthesia 4 hour after the final drug treatment on day 9 as described above; pieces of the livers were collected and transferred immediately into storage at −30°C for lipid level measurements and at −80°C for gene expression measurements.

### 2.6 Measurement of plasma C4 levels

Plasma C4 levels were measured following the method described in our previous report.^23^ Briefly, 25 μL plasma was mixed vigorously with 10 μL of 7β‐hydroxy‐4‐cholesten‐3‐one (50 μg·mL^−1^ in dimethylsulfoxide; Calbiochem, USA), as an internal standard, and 500 μL acetonitrile, and then the mixture was sonicated for 5 minutes. After centrifugation, 100 μL supernatant was processed using a HPLC system (ClassVP; Shimadzu, Japan) fitted with a Nova‐Pak C18 steel column (Waters Corporation, USA), and analyzed with 95% acetonitrile (flow rate: 1 mL·min^−1^) as the mobile phase. The absorbance of C4 was detected at 241 nm; absorbance areas of C4 and the internal standard were calculated, and the concentrations of C4 were quantified against an internal standard.

### 2.7 Blood biochemistry

Plasma total cholesterol and triglyceride (TG) levels were measured enzymatically with a biochemical autoanalyzer (Autoanalyzer type 7070; Hitachi, Tokyo, Japan). For the measurement of HDL‐cholesterol levels, the HDL fraction was separated from apolipoprotein B‐containing lipoproteins by chemical precipitation with heparin and Mn^2+^, and HDL‐cholesterol values were also determined enzymatically using the autoanalyzer. Non‐HDL‐cholesterol values were calculated from differences between the total cholesterol and HDL‐cholesterol values. Post‐treatment values were normalized to the respective pretreatment values and expressed as the percentage of change compared with the control group. To analyze the lipoprotein subclass profiles, hamsters were sacrificed under anesthesia 4 hour after the final drug administration on day 9, and blood samples were collected from the abdominal aorta. After centrifugation, the obtained plasma samples were analyzed using LipoSEARCH technology (Skylight Biotech, Akita, Japan), in which plasma lipoproteins were fractionated by HPLC into 20 subfractions according to particle size.

### 2.8 Measurement of fecal total bile acid and lipid levels

Feces were dried for 6 hour at 90°C. For fecal total bile acid measurement, dried feces (approximately 0.2 g) were homogenized in distilled water (2 mL) and then shaken at 37°C for 15 minutes after the addition of *t*‐butanol (2 mL). After centrifugation twice, the supernatants were pooled and transferred to new tubes by each sample, which were then ready for measurement. Total bile acid values in the samples were measured using an enzymatic colorimetric assay kit (Total Bile Acids Test Wako; Wako Pure Chemical Industries, Ltd, Osaka, Japan). For fecal lipid measurement, dried feces (approximately 1.0 g) was homogenized in a 4 mL of hexane‐isopropanol (3:2) solution and then shaken for 10 minutes at room temperature. The supernatants from two centrifugation steps were pooled, evaporated to dryness, and solidified at 50°C in a nitrogen atmosphere to form a dried residue. After the residues were dissolved in a 1 mL of dioxane‐polyoxyethylene (1:1) solution, the samples were prepared for measurement. Total cholesterol and TG values in the samples were measured using the enzymatic colorimetric assay kits, Cholesterol‐E Test Wako and Triglyceride‐E Test Wako (Wako Pure Chemical Industries, Ltd), respectively. Fecal total bile acid and lipid values were normalized to each dried fecal weight.

### 2.9 Measurement of hepatic lipid levels

The frozen liver samples (approximately 1.0 g) were homogenized by the addition of 3.35% sodium sulfate solution (9 mL) and shaken for 10 minutes after the addition of a 3:2 solution of hexane‐isopropanol (3:2) solution. Subsequently, 100 μL of the solution was evaporated to dryness and solidified at 50°C in a nitrogen atmosphere to form a dried residue. The residue was dissolved in a 100 μL of dioxane‐polyoxyethylene (1:1) solution, and used for the measurement of total cholesterol, free cholesterol, and TG levels using the enzymatic colorimetric assay kits Cholesterol‐E Test Wako, Free Cholesterol‐E Test Wako, and Triglyceride‐E Test Wako (Wako Pure Chemical Industries, Ltd.) respectively. Hepatic cholesteryl ester levels were calculated from differences between the total cholesterol and free cholesterol values. The obtained hepatic lipid values were normalized to the weight of each liver.

### 2.10 Quantitative real‐time polymerase chain reaction (PCR)

The frozen liver samples (approximately 100 mg) were homogenized in ISOGEN solution (Nippon Gene Co., Ltd, Tokyo, Japan) and total RNA was extracted and purified using the RNeasy Mini Kit (QIAGEN K.K., Tokyo, Japan). mRNA expression levels were measured using a one‐step quantitative PCR with TaqMan EZ RT‐PCR Core Reagents (N808‐0236; Applied Biosystems™, Thermo Fisher Scientific K.K.). The primers for hamster CYP7A1 gene (Assay ID: HAMCYP7A1‐EX5) were designed and synthesized based on cDNA sequence using the Assay‐by‐design service (Applied Biosystems™, Thermo Fisher Scientific K.K.). The primers for hamster GAPDH gene (hamster G3PDH‐683F and hamster G3PDH‐748R) were purchased from Sigma‐Aldrich Japan and used as the reference gene. PCR reactions were performed using OPTICON2 (Bio‐Rad Laboratories, Inc., Tokyo, Japan) and gene expression was normalized to GAPDH expression.

### 2.11 Measurement of apolipoprotein A‐I secretion levels in the human hepatoma Huh‐7 cell line

Huh‐7 cells (RCB1366; Riken, Saitama, Japan) were seeded in 24‐well plates in DMEM (Gibco, Thermo Fisher Scientific K.K.) supplemented with 10% FBS (Thermo Fisher Scientific K.K.) and penicillin‐streptomycin solution (Gibco, Thermo Fisher Scientific K.K.) and maintained at 37°C in an atmosphere of 5% CO_2_. Huh‐7 cells under subconfluent conditions were incubated with DMEM supplemented without or with CDCA (final concentration: 30 μmol·L^−1^) and compound‐T1 (final concentration: 0.01‐10 nmol·L^−1^), for 24 hour. Cells that were not treated with CDCA or compound‐T1 were regarded as the control cells. Each well was washed and further incubated for 24 hour under the same conditions. Then, the supernatants were collected and stored at −30°C prior to measurement of apolipoprotein A‐I levels; and cells were dissolved into 0.5 N NaOH and stored at 4°C for prior to the measurement of cellular protein content using the DC protein assay kit (Bio‐Rad Laboratories, K.K., Tokyo, Japan). Apolipoprotein A‐I levels were measured using a sandwich ELISA method. Briefly, the supernatant sample or apolipoprotein A‐I standard solution was added to a 96‐well plate precoated with goat anti‐human apolipoprotein A‐I polyclonal antibodies (Rockland Immunochemicals Inc.; PA, USA) and incubated for 2 hour at room temperature. After washing, rabbit anti‐human apolipoprotein A‐I polyclonal antibodies (Calbiochem, EMD Millipore, Merck KGaA, Darmstadt, Germany) were added and incubated for 2 hour at room temperature. After washing, rabbit IgG specific horseradish peroxidase (HRP)‐conjugated antibodies were added and incubated for 2 hour at room temperature; then, HRP‐derived chemiluminescence was detected using an HRP color development kit (Sumitomo Bakelite Co., Ltd, Tokyo, Japan). The obtained concentrations of apolipoprotein A‐I were normalized to the cellular protein content, and expressed as a percentage relative to the level of control cells.

### 2.12 Statistical analysis

Data are presented as mean ± SEM and were used to perform statistical analyses by the Student's *t*‐test, the Welch's *t*‐test, the one‐tailed Williams' test, or the one‐tailed Shirley‐Williams test. *P* values of ≤.05 were considered significant for the Student's *t*‐test and the Welch's *t*‐test, and ones of ≤.025 were considered significant for the other two tests, The 25% and 50% effective doses and 50% inhibitory concentration were calculated using a nonlinear logistic model.

## 3 RESULTS

### 3.1 FXR antagonistic activity of compound‐T1

Compound‐T1 inhibited CDCA‐induced FXR activation with an IC_50_ value of 2.1 nmol·L^−1^ (Table 1). It did not show agonistic and antagonistic activities against other nuclear receptors related to hepatic lipid metabolism (Table 1), which suggested that compound‐T1 was a potent and selective FXR antagonist.

**Table 1:** Selectivity of compound‐T1 for human nuclear receptors

Antagonistic activities	IC_50_ values (nmol·L^−1^)	Agonistic activities	ED_50_ values (nmol·L^−1^)
FXR	2.1	FXR	>10 000
LXRα	>10 000	LXRα	>10 000
LXRβ	>10 000	LXRβ	>10 000
RXRα	>10 000	RXRα	>10 000
RXRβ	>10 000	PPARα	>10 000
		PPARδ	>10 000
		PPARγ	>10 000

The selectivity of compound‐T1 for human nuclear receptors was measured as described in the Methods section.

### 3.2 CYP7A1 activation by compound‐T1 in a dyslipidemic hamster model

**Figure 2:**
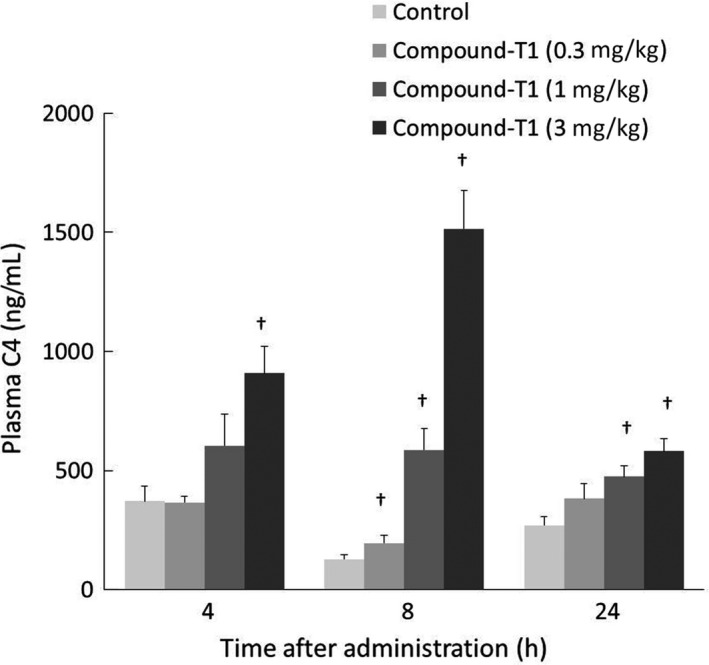
Effects of compound‐T1 on plasma C4 levels. Time‐dependent changes in hepatic gene expression of plasma C4 were measured in samples collected after a single administration of compound‐T1 to high‐fat diet‐fed hamsters. Each value represents the mean ± SEM (n = 6). The measurement procedures are described in the Methods section. Statistical analysis was carried out using one‐tailed Williams' test (^†^ *P* ≤ .025 vs control)

FXR directly activated the expression of short heterodimer partner 1 (SHP‐1), which binds to, and subsequently inactivates, liver receptor homolog 1, and results in the inhibition of CYP7A1 expression. In accordance with this pathway, significant elevation of plasma levels of C4, which is a plasma marker of hepatic CYP7A1 activation, were observed in hamsters receiving an oral dose of compound‐T1; namely, compound‐T1 showed a dose‐dependent increase in plasma C4 levels and sustained the effect for over 24 hours at doses of 1 and 3 mg·kg^−1^ (Figure 2). Based on the result, we considered that an appropriate dose range of compound‐T1 would be greater than 1 mg·kg^−1^·day^−1^ in comparative evaluation of compound‐T1 and the other agents.

### 3.3 Comparative studies on plasma lipid profiles in a dyslipidemic hamster model

**Figure 3:**
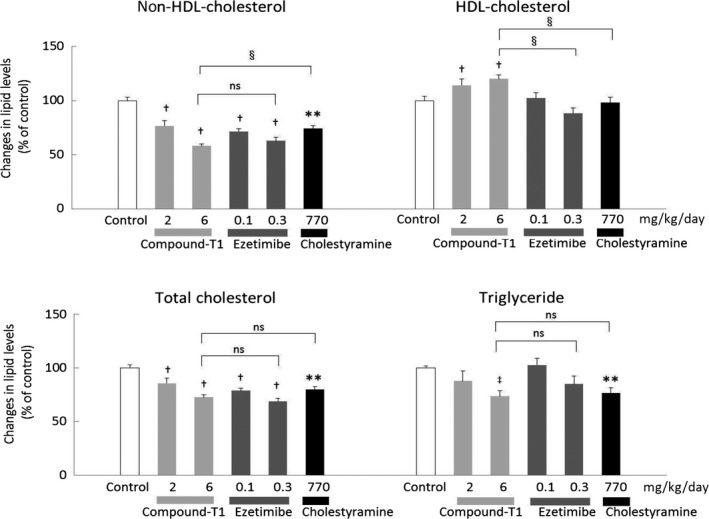
Effects of compound‐T1, ezetimibe, and cholestyramine on plasma lipid parameters in high‐fat diet‐fed hamsters. The changes in lipid parameters were measured in plasma collected after repeated administrations of the treatment drugs. For the control group treated with vehicle, plasma values of non‐HDL‐cholesterol, HDL‐cholesterol, total cholesterol, and triglyceride were 184.0 ± 5.5, 56.0 ± 2.3, 240.0 ±6.9, and 467.5 ± 7.9 mg·dL^−1^ respectively. Each value represents the mean ± SEM (n = 6). The measurement procedures are described in the Methods section. Statistical analysis was carried out using Student's *t*‐test (**P ≤ .01 vs control, or ^§^ *P* ≤ .01 or ns, nonsignificant vs 6 mg·kg^−1^·day^−1^ of compound‐T1), one‐tailed Williams' test (^†^ *P* ≤ .025 vs control), or one‐tailed Shirley‐Williams test (^‡^ *P* ≤ .025 vs control)

Firstly, we conducted a comparative study of compound‐T1 with two cholesterol‐lowering agents, ezetimibe and cholestyramine, in the hamster model. The changes in plasma parameters are summarized in Figure 3 (see also Table S1). Expectedly, these three agents reduced non‐HDL‐cholesterol to the almost same level. Compound‐T1 significantly lowered non‐HDL‐cholesterol, and significantly elevated HDL‐cholesterol. A significant reduction in TG was also observed in the 6 mg·kg^−1^·day^−1^ compound‐T1‐treatment group. Ezetimibe significantly lowered non‐HDL‐cholesterol, but did not change either of HDL‐cholesterol and TG levels. The administration dose of cholestyramine was calculated from the content in the feed and the average food consumption during the first 7 days of drug administration, and was shown to be 770 mg·kg^−1^·day^−1^, which was approximately equal to 6.3 g·day^−1^ (data not shown). The dosing of cholestyramine lowered both non‐HDL‐cholesterol and TG levels but did not affect the HDL‐cholesterol level. No treatments affected food intake and body weight throughout the experimental period (data not shown).

**Figure 4:**
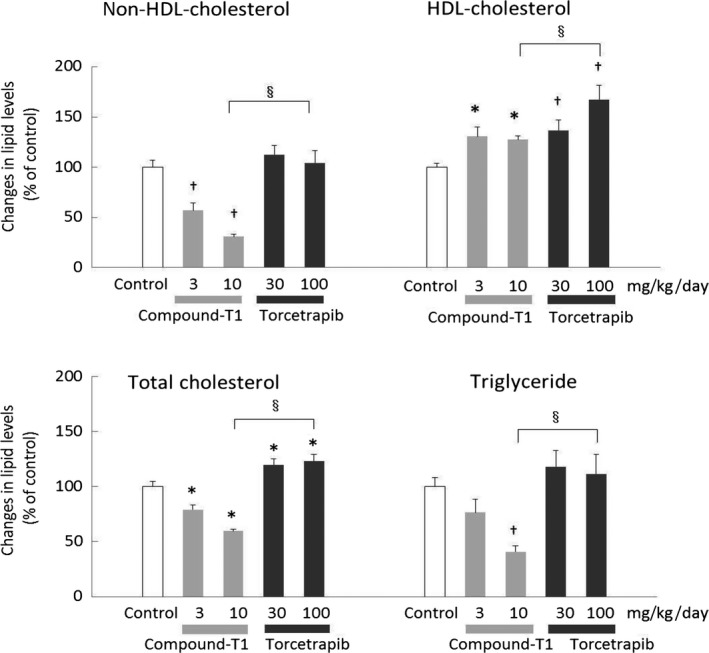
Effects of compound‐T1 and torcetrapib on plasma lipid parameters in high‐fat diet‐fed hamsters. The changes in lipid parameters were measured in plasma collected after repeated administrations of the treatment drugs. For the control group treated with the 10.3% butter diet and no drug, plasma values of non‐HDL‐cholesterol, HDL‐cholesterol, total cholesterol, and triglyceride were 191.1 ± 14.1, 65.3 ± 2.5, 256.4 ± 12.5, and 572.6 ± 46.7 mg·dL^−1^ respectively. Each value represents the mean ± SEM (n = 6). The measurement procedures are described in the Methods section. Statistical analysis was carried out using Welch's *t*‐test (^§^ *P* ≤ .05 vs 10 mg·kg^−1^·day^−1^ of compound‐T1), one‐tailed Williams' test (^†^ *P* ≤ .025 vs control) or one‐tailed Shirley‐Williams test (*P ≤ .025 vs control)

The next study compared compound‐T1 and torcetrapib in the hamster model. Compound‐T1 was dosed at slightly higher levels and was expected to cause HDL‐cholesterol elevation equivalent to torcetrapib. The changes in plasma parameters are summarized in Figure 4 (see also Table S2). Torcetrapib significantly elevated HDL‐cholesterol level but did not lower non‐HDL‐cholesterol and TG levels, which was in contrast to compound‐T1. Although no changes in body weight were observed in all groups, a significant reduction in food intake was observed in the 10 mg·kg^−1^·day^−1^ of compound‐T1‐treated group (−22.5%, data not shown).

**Figure 5:**
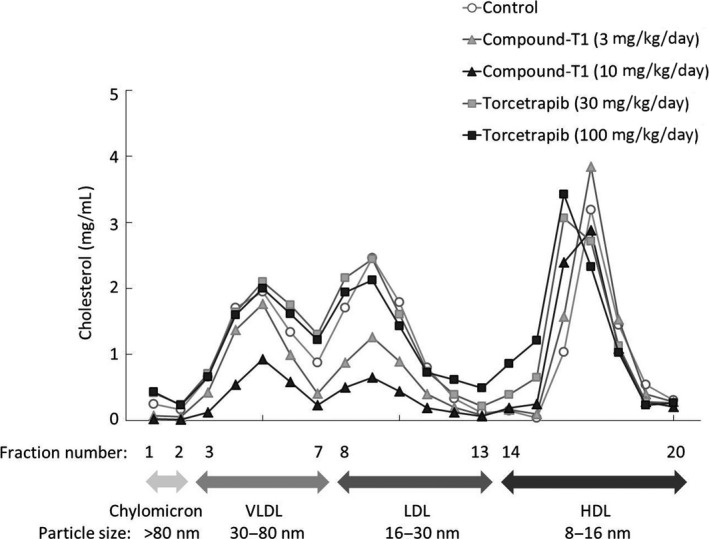
Effects of compound‐T1 and torcetrapib on plasma lipoprotein distribution in high‐fat diet‐fed hamsters. Pooled plasma (n = 6 per group) was collected after repeated drug administrations and then was fractionated by gel filtration HPLC. The measurement procedures are described in the Methods section

To perform a detailed comparison between the HDL‐cholesterol‐elevating effects of compound‐T1 and torcetrapib, we conducted lipoprotein profiling by HPLC gel filtration system (Figure 5). Compound‐T1 dose‐dependently reduced the levels of both very low‐density lipoprotein (VLDL) and low‐density lipoprotein (LDL) with no change in the peak value of particle size. At doses of 3 mg·kg^−1^·day^−1^ and 10 mg·kg^−1^·day^−1^, compound‐T1 also increased HDL levels with no change in the peak value of particle size, but an increase larger HDL particles (fraction number 16) was observed, especially at the higher treatment concentration. In contrast, torcetrapib produced a more marked increase in HDLs with a larger particle diameter; in particular, a dose of 100 mg·kg^−1^·day^−1^ of torcetrapib resulted in an increase in much larger particles (fraction number 13‐15), which were hardly observed in the control and compound‐T1‐treated groups. Torcetrapib did not produce any changes in the VLDL and LDL fractions.

### 3.4 Comparative studies on fecal and hepatic lipid profiles in a dyslipidemic hamster model

**Figure 6:**
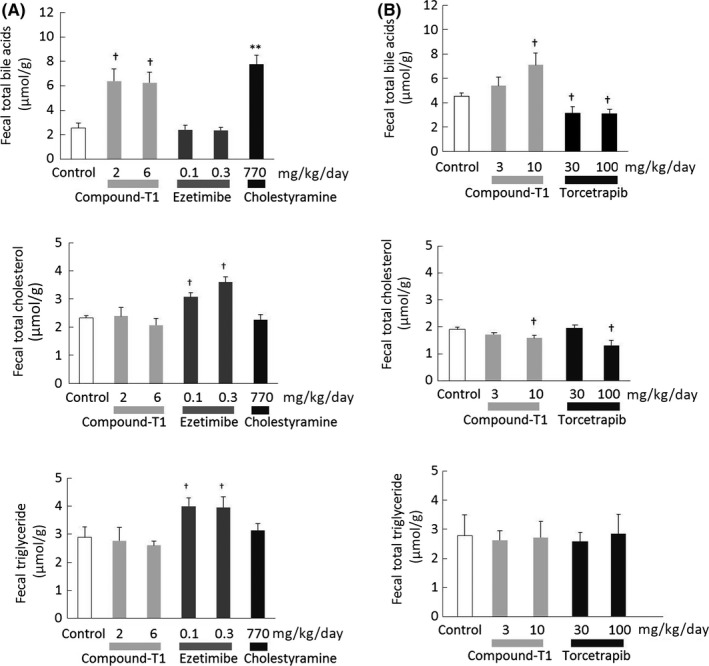
Effects of compound‐T1, ezetimibe, cholestyramine, and torcetrapib on fecal lipid contents in high fat diet‐fed hamsters. Fecal lipid contents were measured in feces collected after repeated drug administration. Comparisons between compound‐T1, ezetimibe, and cholestyramine (A) and between compound‐T1 and torcetrapib (B) were conducted. Each value represents the mean ± SEM (n = 6). The measurement procedures are described in the Methods section. Statistical analysis vs the control was carried out using Student's *t*‐test (**P ≤ .01 vs control) or one‐tailed Williams' test (^†^ *P* ≤ .025 vs control)

The changes in fecal lipid content after treatment with compound‐T1, ezetimibe, and cholestyramine are summarized in Figure 6A. Both compound‐T1 and cholestyramine significantly increased fecal total bile acid content, while ezetimibe increased both fecal total cholesterol and TG content. No other significant changes were observed in all groups.

Next, the changes in fecal lipid content were compared between compound‐T1 and torcetrapib, and are summarized in Figure 6B. Compound‐T1 dose‐dependently increased fecal total bile acid content, while torcetrapib resulted in a significant decrease. A significant reduction in fecal total cholesterol was observed in both groups receiving the highest dosage of compound‐T1 (10 mg·kg^−1^·day^−1^) and torcetrapib (100 mg·kg^−1^·day^−1^). There was no significant change in fecal TG in all groups.

**Figure 7:**
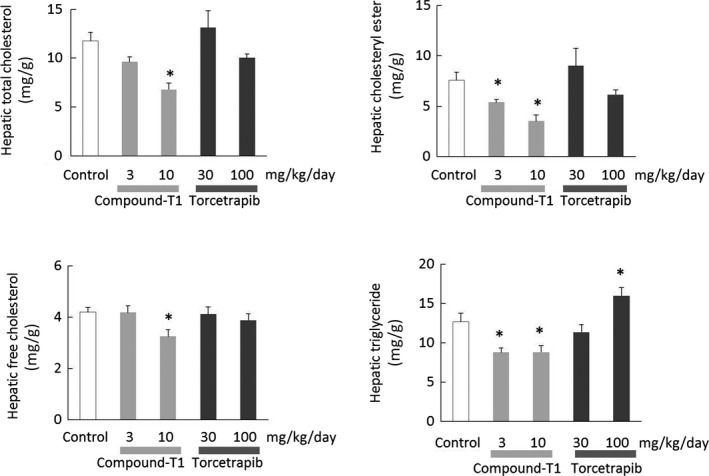
Effects of compound‐T1 and torcetrapib on hepatic lipid content in high‐fat diet‐fed hamsters. Hepatic lipid contents were measured in livers collected after repeated drug administrations. A comparison between compound‐T1‐ and torcetrapib‐treated hamsters was conducted. Each value represents the mean ± SEM (n = 6). The measurement procedures are described in the Methods section. Statistical analysis vs the control was carried out using one‐tailed Williams' test (*P ≤ .025 vs control)

The effects of compound‐T1, ezetimibe, and cholestyramine on hepatic lipid content are summarized in Figure 7. Compound‐T1 dose‐dependently lowered hepatic total cholesterol and cholesteryl ester. Compound‐T1 also decreased hepatic free cholesterol at a dose of 10 mg·kg^−1^·day^−1^. Torcetrapib did not show any significant change in hepatic total cholesterol, cholesteryl ester, and free cholesterol content. In addition, hepatic TG content was decreased in compound‐T1‐treated group, but increased in the 100 mg·kg^−1^·day^−1^ torcetrapib‐treatment group.

**Figure 8:**
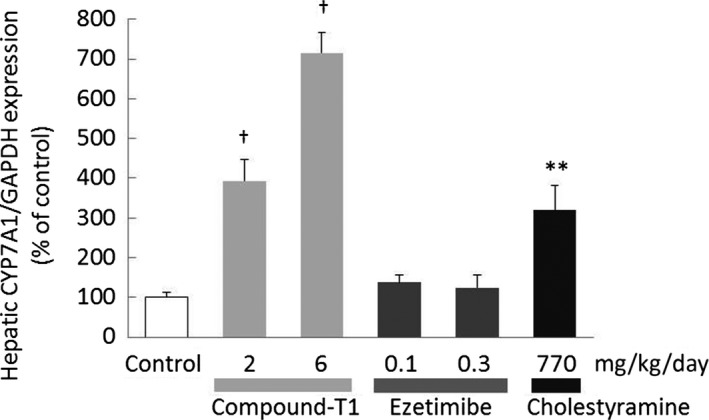
Effects of compound‐T1, ezetimibe, and cholestyramine on hepatic CYP7A1 expression in high‐fat diet‐fed hamsters. Hepatic CYP7A1 expression levels were measured in livers collected after repeated administration of compound‐T1, ezetimibe, and cholestyramine. Each value represents the mean ± SEM (n = 6). The measurement procedures are described in the Methods section. Statistical analysis vs the control was carried out using Student's *t*‐test (**P ≤ .01 vs control) or one‐tailed Shirley‐Williams test (^†^ *P* ≤ .025 vs control)

To confirm the FXR antagonistic activities of compound‐T1 after multiple doses, we measured the hepatic expression levels of CYP7A1. Hepatic CYP7A1 mRNA levels were dose‐dependently increased in the compound‐T1‐treated groups and in the cholestyramine‐treated group. There were no significant changes in the ezetimibe‐treated groups (Figure 8).

### 3.5 Effects of compound‐T1 on apolipoprotein A‐I production in Huh‐7 cells

**Figure 9:**
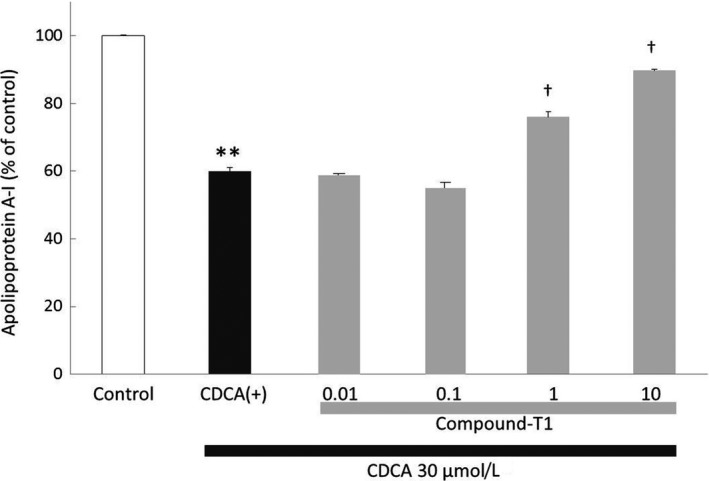
Effect of compound‐T1 on apolipoprotein A‐I secretion in CDCA‐treated Huh‐7 cells. Apolipoprotein A‐I concentration in cell supernatant was measured with a sandwich ELISA method, as described in the Methods section. Each value represents the mean ± SEM (n = 3). Statistical analysis was carried out using Student's *t*‐test (**P ≤ .01 vs control) or one‐tailed Williams' test (^†^ *P* ≤ .025 vs CDCA(+))

To elucidate the mechanism of the HDL‐cholesterol‐elevating effects of compound‐T1, we investigated its effect on apolipoprotein A‐I production using the human hepatoma Huh‐7 cell line under physiological CDCA conditions. Apolipoprotein A‐I secretion was significantly reduced by the addition of 30 μmol·L^−1^ CDCA, and this reduction was dose‐dependently rescued by compound‐T1 with an ED_50_ value of 1.8 nmol·L^−1^ (Figure 9).

## 4 DISCUSSION

We have previously confirmed that a synthesized FXR antagonist, compound‐T3, reduced plasma non‐HDL‐cholesterol levels and also elevated HDL‐cholesterol levels in a primate model.^21^, ^22^ However, the development of a rodent model with similar disease conditions to human hyperlipidemia would be still useful for the further comprehension of this new type of agent. However, the rodent animals such as mice, rats, and guinea pigs have different plasma lipid and lipoprotein profiles from humans.^23^, ^25^ Actually, we found that another FXR antagonist, compound‐T0, exacerbated dyslipidemia in mice due to enhancement of intestinal lipid absorption via acceleration of bile acid excretion.^26^ We think that the difference of the effects of FXR antagonist between primate and mouse models would be due to the difference in regulation of key players relating to lipid and bile acids metabolisms, in particular, the hepatic LDL receptor.^27^ In addition, CETP is also a key protein involved in plasma cholesterol transport that transfers cholesteryl ester from HDL to LDL and VLDL.^28^ Golden Syrian hamsters are known to develop human‐like hyperlipidemia following the feeding of a high‐fat diet,^29^, ^30^, ^31^ and also show both plasma CETP activity^28^ and regulation of hepatic LDL receptor.^32^ Therefore, we considered that a hyperlipidemic hamster model would be suitable for the purpose described above. To determine the appropriate diet for hamsters, we referenced diet information in nonclinical and clinical studies of orlistat, an antiobesity drug.^33^, ^34^ After the hamsters were fed this butter‐rich diet for a week, both plasma levels of cholesterol and triglyceride increased to approximately two and three times the normal level respectively (Table S3). Using this hamster model, herein, we sought to clarify the favorable pharmacological profile of a novel FXR antagonist compound‐T1, a potential antidyslipidemic agent, through comparative studies with other nonstatin agents ezetimibe, cholestyramine, and torcetrapib.

The first question in our study was to determine whether the clinical results of other nonstatin agents were consistent in our hamster model. Firstly, ezetimibe, a cholesterol absorption inhibitor, exhibited clinically relevant changes with regard to the plasma non‐HDL‐cholesterol reduction and fecal cholesterol excretion.^35^, ^36^ Although a significant increase in fecal TG was also found in ezetimibe‐treated hamsters, as reported in a previous study using an obese rodent model,^37^ the dramatic effect of ezetimibe on TG excretion has not been reported in a clinical setting. However, an inhibitory effect on the intestinal production of chylomicrons, which consist largely of TG, was reported in patients with hyperlipidemia.^38^ Therefore, we assumed that the increased fecal TG in ezetimibe‐treated hamsters might result from its intervention in chylomicron TG metabolism. In the next, cholestyramine, a bile acid sequestrant, reduced plasma non‐HDL‐cholesterol at a dose of 770 mg·kg^−1^·day^−1^ (approximately equivalent to 6.3 g·day^−1^). This is a clinically sufficient dose; specifically, a dose of 8‐24 g·day^−1^ of cholestyramine is usually set for hyperlipidemia patients.^39^, ^40^ Cholestyramine also elevated hepatic CYP7A1 expression and fecal total bile acid content, which suggested the mechanism of action occurred through the activation of the cholesterol catabolism pathway. In addition, the CETP inhibitor torcetrapib resulted in a remarkable increase in HDL‐cholesterol, similar to those observed in clinical trials. In the present study, torcetrapib did not induce changes in either of fecal cholesterol and bile acid contents, which suggested that torcetrapib did not increase the overall amount of lipid excreted out of the body. The analysis of plasma lipoprotein subclass revealed that torcetrapib increased the fraction of HDL with a larger particle diameter. Although these findings were consistent with clinical observations,^41^, ^42^ the significance of large HDL particles has not been determined. However, it was reported that subjects with a genetic CETP deficiency, who are expected to have high levels of larger HDLs,^43^ had an increase in coronary heart disease in some analysis.^44^, ^45^ The large HDL particles warrant further investigation both subfractionally and functionally.

As discussed above, our hamster model reflected the most of clinical responses of nonstatin agents; therefore, the next question was to determine how the FXR antagonist exhibited its antidyslipidemic effects. In this model, compound‐T1 significantly lowered plasma non‐HDL‐cholesterol and elevated plasma HDL‐cholesterol. FXR is reported to regulate the expression genes involved in SHP‐1 and apolipoprotein A‐I in the liver; in particular, the upregulation of SHP‐1 by FXR decreases the expression of CYP7A1.^19^, ^20^ This was consistent with the compound‐T1‐induced changes observed in this study: elevated hepatic expression of CYP7A1 decreased hepatic cholesterol and increased fecal bile acids. It was suggested that compound‐T1 accelerated both the catabolism of cholesterol to bile acids and their excretion into feces. Plasma TG levels were significantly decreased in hamsters that received 6 mg·kg^−1^·day^−1^ compound‐T1 or greater, but it was assumed that the higher doses constitute an excessive dose, because a reduction in food intake (‐22.5%) was observed in the group receiving a dose of 10 mg·kg^−1^·day^−1^. In addition, in human hepatoma Huh‐7 cells, compound‐T1 concentration‐dependently rescued the CDCA‐induced reduction in apolipoprotein A‐I production, and the ED_50_ value was 1.8 nmol·L^−1^, which was a similar concentration to the IC_50_ value in the human FXR antagonism reporter gene assay (Table 1). Additionally, when an oral dose of 1.0 mg·kg^−1^ compound‐T1 was administered to hamsters fed a standard diet, the maximum plasma concentration was 12.4 nmol·L^−1^ and the AUC_0‐24 h_ liver:plasma ratio was approximately 400:1 (data not shown). Despite the consideration of interspecies differences, it was expected that the required amount of compound‐T1 to enhance apolipoprotein A‐I production would be available in the livers of the compound‐T1‐treated hamsters in the present study. To the best of our knowledge, this is the first pharmacological evaluation of an FXR antagonist in a dyslipidemic hamster model.

The final question addressed in this study was whether the FXR antagonist possessed advantages over other nonstatin agents. Based on our findings described above, it has been suggested that the FXR antagonist could uniquely modulate both non‐HDL‐cholesterol and HDL‐cholesterol metabolism. Specifically, ezetimibe exhibited a large reduction in plasma non‐HDL‐cholesterol, but as discussed above, its mechanism was unrelated to the one of compount‐T1. In contrast, cholestyramine and compound‐T1 acted through similar mechanisms; both agents activated the cholesterol catabolism pathway. However, cholestyramine required a relatively high dose to confer a sufficient cholesterol‐lowering effect; this was also consistent with clinical observations as described above. A high dosage requirement can sometimes affect patient drug compliance.^46^ In this respect, compound‐T1 would be therefore superior to cholestyramine. Additionally, in the present study, compound‐T1 did not result in a large change in HDL particle size, but torcetrapib did. Future studies are needed to clarify this, but it would be important to investigate whether increased HDL particles can promote the reverse cholesterol transport system to cause cholesterol efflux from atherosclerotic lesions to the liver and feces; in particular, we recommend a focus on apolipoprotein A‐I, which has been reported to be a key factor in the effective promotion of beneficial HDL metabolism.^47^ Indeed, intravenous infusion of a variant of apolipoprotein A‐I‐phospholipid complexes resulted in the regression of atherosclerosis in patients with acute coronary syndromes.^48^ In this regard, increased antiatherosclerotic effects may be expected from compound‐T1, which directly upregulated apolipoprotein A‐I. The functional differences in the increased HDL particles might create distinct clinical outcomes between both agents.

Generally, the high‐fat diet‐fed hamster model was a good reflection of the clinical results of nonstatin agents including ezetimibe, cholestyramine, and torcetrapib, which emphasized the benefits of utilizing hamsters as a rodent model of human‐like dyslipidemia. More importantly, our study demonstrated that compound‐T1, a novel selective FXR antagonist, induced remarkable beneficial changes in both plasma non‐HDL‐cholesterol and HDL‐cholesterol levels in the hamster model, and that these effects were thought to involve the enhancement of cholesterol catabolism and apolipoprotein A‐I production. Our results suggested that FXR antagonists have the potential to become a novel class of antidyslipidemic agents that offer the unique regulation of both lipid and bile acid metabolism.

## AUTHOR CONTRIBUTIONS

E. Shinozawa and Y. Amano designed and performed the experiments, analyzed and interpreted data, and wrote the manuscript. H. Yamakawa and M. Haba designed and performed the experiments, and analyzed and interpreted data. M. Shimada prepared the compound and supervised the study. R. Tozawa supervised the study and wrote the manuscript.

## DISCLOSURE

The authors are employees of Takeda Pharmaceutical Co., Ltd (Osaka, Japan) at which Compound‐T1 was synthesized.
