# Supplementary material for: Antidyslipidemic potential of a novel farnesoid X receptor antagonist in a hamster model of dyslipidemia: Comparative studies of other nonstatin agents
Source: Pharmacol Res Perspect. 2018 Mar 8;6(2):e00390. doi: 10.1002/prp2.390 (PMC5842406; doi:10.1002/prp2.390)
Supplement: Supplementary file 1 [file PRP2-6-e00390-s001.docx]

Supplemental Table S1: Effects of compound-T1, ezetimibe, and cholestyramine on plasma lipid parameters in high-fat diet-fed hamsters.

| Group | | Total cholesterol | | | |
| --- | --- | --- | --- | --- | --- |
|  | | initial  (mg dL^-1^) | after dosing (mg dL^-1^) | % of initial | % of control |
| Control | mean | 240.1 | 240.0 | 100.3 | 100.0 |
|  | SEM | 7.2 | 6.9 | 3.6 | 2.9 |
| Compound-T1 | mean | 248.1 | 205.3 | 82.6 | 85.6 |
| 2 mg kg^-1^ day^-1^ | SEM | 4.6 | 11.2 | 3.5 | 4.7 |
| Compound-T1 | mean | 245.8 | 174.3 | 71.2 | 72.6 |
| 6 mg kg^-1^ day^-1^ | SEM | 7.4 | 4.8 | 2.8 | 2.0 |
| Ezetimibe | mean | 239.9 | 188.8 | 78.9 | 78.7 |
| 0.1 mg kg^-1^ day^-1^ | SEM | 7.0 | 5.4 | 2.6 | 2.3 |
| Ezetimibe | mean | 240.6 | 165.3 | 68.9 | 68.9 |
| 0.3 mg kg^-1^ day^-1^ | SEM | 4.8 | 5.9 | 2.9 | 2.5 |
| Cholestyramine | mean | 248.3 | 191.5 | 77.2 | 79.8 |
| 770 mg kg^-1^ day^-1^ | SEM | 6.4 | 6.7 | 2.2 | 2.8 |
| Group | | Triglyceride | | | |
|  | | initial  (mg dL^-1^) | after dosing (mg dL^-1^) | % of initial | % of control |
| Control | mean | 358.6 | 467.5 | 137.9 | 100.0 |
|  | SEM | 41.0 | 7.9 | 13.4 | 1.7 |
| Compound-T1 | mean | 355.6 | 409.9 | 122.0 | 87.7 |
| 2 mg kg^-1^ day^-1^ | SEM | 38.6 | 44.7 | 16.9 | 9.6 |
| Compound-T1 | mean | 357.3 | 343.0 | 106.3 | 73.4 |
| 6 mg kg^-1^ day^-1^ | SEM | 41.8 | 31.1 | 22.5 | 6.7 |
| Ezetimibe | mean | 369.4 | 480.3 | 137.4 | 102.7 |
| 0.1 mg kg^-1^ day^-1^ | SEM | 37.1 | 29.2 | 17.2 | 6.2 |
| Ezetimibe | mean | 345.5 | 396.3 | 116.2 | 84.8 |
| 0.3 mg kg^-1^ day^-1^ | SEM | 31.0 | 35.3 | 9.2 | 7.5 |
| Cholestyramine | mean | 348.8 | 357.5 | 111.1 | 76.5 |
| 770 mg kg^-1^ day^-1^ | SEM | 47.8 | 23.5 | 14.3 | 5.0 |
| Group | | non-HDL-cholesterol | | | |
|  | | initial  (mg dL^-1^) | after dosing (mg dL^-1^) | % of initial | % of control |
| Control | mean | 170.9 | 184.0 | 108.0 | 100.0 |
|  | SEM | 6.2 | 5.5 | 3.1 | 3.0 |
| Compound-T1 | mean | 177.0 | 141.3 | 79.6 | 76.8 |
| 2 mg kg^-1^ day^-1^ | SEM | 4.3 | 8.5 | 3.5 | 4.6 |
| Compound-T1 | mean | 177.2 | 107.1 | 60.7 | 58.2 |
| 6 mg kg^-1^ day^-1^ | SEM | 5.4 | 4.3 | 3.0 | 2.3 |
| Ezetimibe | mean | 167.6 | 131.6 | 79.0 | 71.5 |
| 0.1 mg kg^-1^ day^-1^ | SEM | 5.9 | 4.8 | 4.1 | 2.6 |
| Ezetimibe | mean | 168.8 | 115.9 | 69.1 | 63.0 |
| 0.3 mg kg^-1^ day^-1^ | SEM | 5.0 | 6.1 | 4.7 | 3.3 |
| Cholestyramine | mean | 179.8 | 136.4 | 76.2 | 74.1 |
| 770 mg kg^-1^ day^-1^ | SEM | 6.5 | 4.9 | 3.1 | 2.6 |
| Group | | HDL-cholesterol | | | |
|  | | initial  (mg dL^-1^) | after dosing (mg dL^-1^) | % of initial | % of control |
| Control | mean | 69.2 | 56.0 | 81.7 | 100.0 |
|  | SEM | 3.5 | 2.3 | 5.0 | 4.2 |
| Compound-T1 | mean | 71.1 | 64.0 | 90.3 | 114.4 |
| 2 mg kg^-1^ day^-1^ | SEM | 2.2 | 3.1 | 4.8 | 5.6 |
| Compound-T1 | mean | 68.6 | 67.2 | 98.5 | 120.1 |
| 6 mg kg^-1^ day^-1^ | SEM | 2.3 | 2.3 | 4.3 | 4.1 |
| Ezetimibe | mean | 72.3 | 57.3 | 79.3 | 102.3 |
| 0.1 mg kg^-1^ day^-1^ | SEM | 3.5 | 2.9 | 2.0 | 5.2 |
| Ezetimibe | mean | 71.8 | 49.4 | 68.7 | 88.3 |
| 0.3 mg kg^-1^ day^-1^ | SEM | 1.1 | 2.9 | 3.3 | 5.1 |
| Cholestyramine | mean | 68.5 | 55.1 | 80.2 | 98.4 |
| 770 mg kg^-1^ day^-1^ | SEM | 1.7 | 2.7 | 2.6 | 4.8 |

Supplemental Table S2: Effects of compound-T1 and torcetrapib on plasma lipid parameters in high-fat diet-fed hamsters.

| Group | | Total cholesterol | | | |
| --- | --- | --- | --- | --- | --- |
|  | | initial  (mg dL^-1^) | after dosing (mg dL^-1^) | % of initial | % of control |
| Control | mean | 258.1 | 256.4 | 99.8 | 100.0 |
|  | SEM | 13.0 | 12.5 | 4.3 | 4.9 |
| Compound-T1 | mean | 259.5 | 202.9 | 78.9 | 79.1 |
| 3 mg kg^-1^ day^-1^ | SEM | 11.2 | 10.3 | 5.1 | 4.0 |
| Compound-T1 | mean | 253.6 | 153.4 | 60.4 | 59.8 |
| 10 mg kg^-1^ day^-1^ | SEM | 13.1 | 3.6 | 2.2 | 1.4 |
| Torcetrapib | mean | 256.6 | 307.4 | 120.3 | 119.9 |
| 30 mg kg^-1^ day^-1^ | SEM | 9.8 | 14.1 | 5.9 | 5.5 |
| Torcetrapib | mean | 254.6 | 315.8 | 124.7 | 123.2 |
| 100 mg kg^-1^ day^-1^ | SEM | 12.2 | 15.7 | 6.2 | 6.1 |
| Group | | Triglyceride | | | |
|  | | initial (mg dL^-1^) | after dosing (mg dL^-1^) | % of initial | % of control |
| Control | mean | 424.0 | 572.6 | 141.8 | 100.0 |
|  | SEM | 40.5 | 46.7 | 18.5 | 8.2 |
| Compound-T1 | mean | 447.8 | 439.1 | 97.1 | 76.7 |
| 3 mg kg^-1^ day^-1^ | SEM | 25.5 | 67.5 | 11.7 | 11.8 |
| Compound-T1 | mean | 454.1 | 233.6 | 51.2 | 40.8 |
| 10 mg kg^-1^ day^-1^ | SEM | 8.4 | 32.0 | 7.1 | 5.6 |
| Torcetrapib | mean | 453.8 | 676.1 | 154.2 | 118.1 |
| 30 mg kg^-1^ day^-1^ | SEM | 31.3 | 84.7 | 24.6 | 14.8 |
| Torcetrapib | mean | 453.9 | 638.8 | 146.2 | 111.6 |
| 100 mg kg^-1^ day^-1^ | SEM | 48.3 | 100.1 | 25.6 | 17.5 |
| Group | | non-HDL-cholesterol | | | |
|  | | initial  (mg dL^-1^) | after dosing (mg dL^-1^) | % of initial | % of control |
| Control | mean | 174.9 | 191.1 | 110.0 | 100.0 |
|  | SEM | 10.4 | 14.1 | 8.5 | 7.4 |
| Compound-T1 | mean | 175.3 | 129.3 | 74.6 | 67.7 |
| 3 mg kg^-1^ day^-1^ | SEM | 10.6 | 13.9 | 7.7 | 7.3 |
| Compound-T1 | mean | 169.8 | 72.8 | 42.2 | 38.1 |
| 10 mg kg^-1^ day^-1^ | SEM | 14.4 | 5.3 | 3.0 | 2.8 |
| Torcetrapib | mean | 175.0 | 221.7 | 129.7 | 116.0 |
| 30 mg kg^-1^ day^-1^ | SEM | 12.2 | 17.5 | 13.6 | 9.1 |
| Torcetrapib | mean | 173.8 | 208.9 | 121.5 | 109.3 |
| 100 mg kg^-1^ day^-1^ | SEM | 11.8 | 23.6 | 14.2 | 12.3 |
| Group | | HDL-cholesterol | | | |
|  | | initial  (mg dL^-1^) | after dosing (mg dL^-1^) | % of initial | % of control |
| Control | mean | 83.2 | 65.3 | 80.3 | 100.0 |
|  | SEM | 4.9 | 2.5 | 7.4 | 3.8 |
| Compound-T1 | mean | 84.2 | 73.6 | 87.9 | 112.7 |
| 3 mg kg^-1^ day^-1^ | SEM | 1.7 | 6.2 | 8.1 | 9.5 |
| Compound-T1 | mean | 83.8 | 80.6 | 99.8 | 123.5 |
| 10 mg kg^-1^ day^-1^ | SEM | 3.6 | 2.3 | 4.3 | 3.5 |
| Torcetrapib | mean | 81.6 | 85.7 | 106.8 | 131.2 |
| 30 mg kg^-1^ day^-1^ | SEM | 4.0 | 6.8 | 10.8 | 10.3 |
| Torcetrapib | mean | 80.8 | 106.9 | 133.0 | 163.7 |
| 100 mg kg^-1^ day^-1^ | SEM | 2.7 | 9.2 | 11.5 | 14.1 |

Supplemental Table S3: Plasma lipid levels of hamsters fed with a standard chow diet (CE-2, n=42).

|  | Total cholesterol (mg dL^-1^) | Triglyceride (mg dL^-1^) | non-HDL-cholesterol (mg dL^-1^) | HDL-cholesterol (mg dL^-1^) |
| --- | --- | --- | --- | --- |
| mean | 151.6 | 122.4 | 105.9 | 45.7 |
| SEM | 0.9 | 4.0 | 0.9 | 0.5 |
